# Academician Andrey Ivanovich Vorobiev, 1 November 1928 to 15 June 2020: founder of modern Soviet/Russian haematology

**DOI:** 10.1038/s41375-020-0985-8

**Published:** 2020-07-16

**Authors:** Nikita E. Shklovskiy-Kordi, Robert Peter Gale

**Affiliations:** 1 National Research Center for Hematology, Moscow, Russia; 2 Centre for Haematology, Department of Immunology and Inflammation, Imperial College London, London, UK

**Keywords:** Cancer, Health care


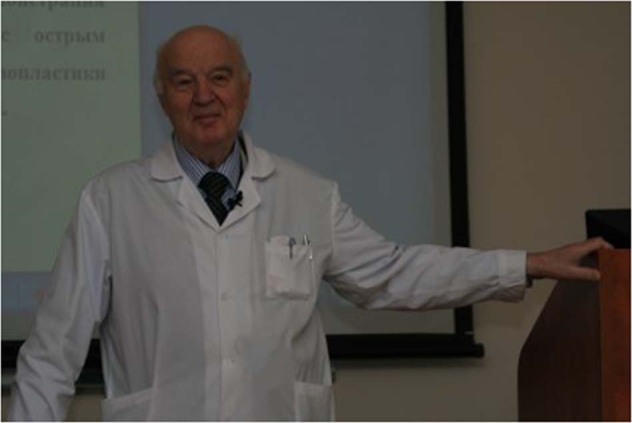


Sometimes we hear of people overcoming extraordinary odds on the way to success. However, few stories compare with that of Academician Andrey Ivanovich Vorobiev, a giant in modern Soviet/Russian haematology. Andrey Vorobiev was born on 1 November 1928 in Moscow. His father, a physician, and his mother, a biologist, were members of the *Trotskist opposition*, an anti-Stalin group in the Bolshevik Party. Andrey’s father was executed in 1936 by the NKVD when Andrey was 8 years old. After 2 weeks, his mother received an 18-year prison sentence and was sent to the Kolyma region in the Soviet Far East, site of the harshest labour camps in the Gulag Archipelago (Aleksandr Solzhenitsyn discusses an uprising in her camp in his book, *The Gulag Archipelago)*. Vorobiev was saved by a nanny (at this time a poor doctor could hire a housekeeper because there were very hungry peasants in the city) and by his grandmother. In 1941, age 13 years, he was sent to an orphanage when children were evacuated in advance of the German siege of Moscow. There he earned his first piece of bread cleaning an outdoor public latrine in −30 °C weather. At age 14, he began working as a painter at a construction site whilst attending night school. During this time, Andrey sent parcels to his mother in prison including onions and dried bread. He bartered bread rations for onions with the local people. Later in life she said: *I owe your* parcels *my life*. Perhaps Vorobiev already had medical insight: onions saved her from scurvy and folic acid, from anaemia. Years later Prof. Rudiger Hehlmann visited a memorial at the Akmola (White Death) prison camp in present day Kazakhstan near Nur-Sultan (Astana) where *wives of traitors of the Motherland* were exiled. He found Andrey’s mothers name listed on the memorial where she apparently saved many lives as the camp paediatrician.

In 1947, Andrey graduated school with a gold medal and entered the 1st Moscow Medical Institute. His admission was a miracle. A friend of his executed father working at the Institute switched application documents so that Andrei did not have to go for an interview. Otherwise, as a son of *enemies of the people* he would never have been admitted. Graduating with honours in 1953 Vorobiev was sent to a rural hospital in Volokolamsk about 130 km north of Moscow where he worked as a general practitioner, paediatrician and pathologist whilst supervising a polyclinic and the premature infant nursery of a nearby maternity hospital. He was able to meet these challenges because his colleagues were supportive, experienced older physicians. He kept this memory of his days in Volokolamsk his entire life and recalled it almost daily.

In 1956, during the Khrushchev thaw Vorobiev began residency training with the famous haematologist Prof. I. A. Kassirsky at the Central Institute of Advanced Medical Studies. Andrey became Chair of Haematology there in 1971, a position he held until 2018. At the Institute he pioneered the calorimetric method for determining haemoglobin concentration.

Therapy of haematological cancers was in its infancy when Vorobiev began his research. In 1972, shortly after Prof. Donald Pinkel from St. Jude and his colleagues published their Total Therapy study of treatment of children with acute lymphoblastic leukaemia (ALL), Vorobiev and coworkers were the first in USSR to use the St. Jude protocol to treat children with ALL. Remarkably, they did their work at the Institute of Biophysics of the USSR Ministry of Health where only adults were to be treated. Also remarkably, they independently invented a three-drug intra-thecal chemotherapy regimen to prevent CNS relapse because they lacked equipment to do CNS radiation therapy, ironic in an Institute dedicated to radiation biology. God knows how they got away with these things.

From 1966 to 1974 Vorobiev headed the clinical department at the Institute where he developed an innovative biological dosimetry method to accurately estimate dose in persons exposed to ionizing radiations. In 1986, he returned to the Institute to manage medical aspects of the Chernobyl nuclear power facility accident. At that time Premier Nikolai Ivanovich Ryzhkov asked Vorobiev if having foreigners in the clinic would compromise the Soviet nuclear programme. Vorobiev’s reply: *We are ready for openness*. Chernobyl became the turning point for the policy of *openness* (glasnost) of Mikhail Sergeyevich Gorbachev, then General Secretary of the Communist Party.

From 1987 to 2011 Vorobiev was Director of the State Hematology Research Centre of the Russian Academy of Medical Sciences which evolved from the Central Institute of Blood Transfusion. Under his direction the Centre was responsible for treating victims of almost all industrial and natural disasters including the Armenia (1988) and Sakhalin (1994) earthquakes. He organised the first Intensive care department specifically for people with blood cancers and specialised in diagnosis and treatment of disseminated intra-vascular coagulation (DIC). Vorobiev lead developments in Russia in lymphoma therapy. Vorobiev was the first Minister of Health of the Russian Federation from 1991 to 1992 where he used his position to save high-technology medicine during the collapse of the Soviet economy. He also used his position for other important tasks. Prof. Dieter Hoelzer reports that on visits to the former West Germany Andrey delighted in excursions to hardware/garden shops looking for items to improve his dacha. On one occasion, he purchased a large axe which, because of his diplomatic passport, he was able to skip security and bring it aboard the plane to Moscow. This, of course, caused alarm amongst the cabin crew who insisted the axe be checked. Andrey resisted arguing it would surely be stolen by the luggage handlers at Sheremetyevo airport. The compromise: The axe stayed in the cockpit for the flight and was returned to Andrey on arrival. Whether he used it on the notoriously inefficient immigration officers on entry is unknown.

As a consultant to the Main Medical Directorate of Government Medicine, Vorobiev took part in the treatment of almost all leaders of the USSR and the Russian Federation from the mid-80s to 2009 including General Secretaries Yuri Vladimirovich Andropov, and President Boris Nikolayevich Yeltsin. Many of his medical decisions were of enormous consequence but, following the Hippocratic Oath, he never divulged his role.

A School of Medicine based on clinical and morphological studies, started in Soviet Union by Profs. A. N. Kryukov and I. A. Kassirsky, was successfully advanced and further developed by Prof. Vorobiev. Even in his final days he examined patients and did his own interpretation of blood and bone marrow slides. He was editor of the main Soviet/Russian Handbook of Hematology, *Atlas of Tumors of the Lymphatic Blood System* (A. I. Vorobyov and A. M. Kremenetskaya eds.).

Because of his achievements, many consider Vorobiev the founder of modern Russian haematology. Most Americans and Europeans may be unaware of the seminal contributions of Vorobiev and his colleagues. This is sadly because of restrictions on the ability of Soviet and Russian scientist, especially those involved in sensitive areas such as radiation biology to publish their research in the Western biomedical literature. A classic and unfortunate case of *don’t publish and perish*.

An anecdote. Immediately after the Chernobyl nuclear power facility accident RPG and his Russian colleagues Profs. Angelina Konstantinovna Guskova and Alexander Evgenivich Baranov wanted to treat the victims with granulocyte/macrophage-colony stimulating factor (G/M-CSF). We had considerable experience with G/M-CSF in rodents and monkeys but none in humans. I convinced Sandoz in Basel to send us some G/M-CSF. At this point Andrey went to the Special Chernobyl Commission of the Politburo to request permission. The commission declined saying they didn’t want Soviet citizens to be *guinea pigs*. We were disappointed but then we devised a solution. We would be the *guinea pigs*. Andrey and I injected each other with what we estimated to be the correct dose based on our data in monkeys. I was reluctant to do as Andrey was almost 60 years old at the time which seemed rather old to me then (how our perspective changes). Feeling fine we parted and left the hospital for the evening. About 2 h later, I received a phone call at Spaso House where I was dining with the American ambassador, Arthur Hartman: *Prof. Vorobiev is dying*. Rushing back to the hospital (believing I would never leave the Soviet Union alive). I found him in the coronary ICU pale, diaphoretic and complaining of chest pain. The electro-cardiogram was normal as were the cardiac enzymes. After some questioning I deduced he had sternal, not chest pain from the G/M-CSF (The monkeys never said anything.). By next morning, our WBCs were sky high and we received permission from the Commission to treat the Chernobyl radiation victims with considerable success. This anecdote illustrates Prof. Vorobiev’ s dedication to his profession and to helping his patients.

Many other colleagues recall incidents revealing Prof. Vorobiev’s outstanding character. For example, Prof. Hans-Jochem Kolb and RPG recall a bus trip from Moscow to Pushkinskije Gory (country residence of Russian poet Alexandre Pushkin) during Soviet times. The bus, with 40 or so distinguished Russian and foreign scientists including members of the Soviet National Academy of Science, was in considerable dis-repair and roads were terrible. About halfway here we ran out of petrol. What to do? Andrey had the driver take us to the nearby home of the Russian composer Modest Petrovich Mussorgsky. Somehow, he arranged for a local piano student to entertain us with Mussorsky’s *Night on Bold Mountain* and *Pictures at an Exhibition* on the compser’s original piano. If this sounds a long programme it’s because of the considerable time needed to find petrol in the middle of nowhere. We were lucky, we might have had to add *Die Meistersinger von Nürnberg* without Andrey’s influence.

Given his background it is not surprising that Prof. Vorobiev was a strong human rights activist. He defended peoples’ right to free medical care and strongly opposed the death penalty. As a Minister of Health of the Russian Federation he also started hospices in Russia whilst simultaneously arguing people with incurable diseases should have the opportunity to receive experimental therapies. Vorobiev was adamant in his hatred of state tyranny. In 1938, 10-year-old Andrey created, with another boy a secret organisation, *Death to Stalin*. Fortunately for him (and us), the organisation was not discovered by the NKVD although Andrei wore an icon with a crossed out “C”. When 74 years later he received a Human Rights Protection Medal from the government of the Russian Federation, he rose from his wheelchair (he’d had a stroke) and said to the amazed audience: *In Russia there is the wide practice of torture. Until this stops results of investigations can’t be believed and there is no justice*. Fortunately, Beria was not in the audience but his successors may have been. But as Andrey confided to RPG: *What can they do to a 90-year-old man?*

Professor Vorobiev is survived by his wife, Dr. Alexandra Kremenetskaya, his sons Profs. Ivan and Pavel Vorobiev and 20 grandchildren. We mourn the loss of a mentor and friend. Russian haematology is the poorer without him.

